# Overview and Empirical Analysis of ISP Parameter Tuning for Visual Perception in Autonomous Driving

**DOI:** 10.3390/jimaging5100078

**Published:** 2019-09-24

**Authors:** Lucie Yahiaoui, Jonathan Horgan, Brian Deegan, Senthil Yogamani, Ciarán Hughes, Patrick Denny

**Affiliations:** Valeo Vision Systems, Dunmore Road, Tuam, Co. Galway H54 Y276, Ireland; jonathan.horgan@valeo.com (J.H.); brian.deegan@valeo.com (B.D.); ciaran.hughes@valeo.com (C.H.); patrick.denny@valeo.com (P.D.)

**Keywords:** image signal processing, computer vision, autonomous driving, localization, reconstruction, recognition

## Abstract

Image quality is a well understood concept for human viewing applications, particularly in the multimedia space, but increasingly in an automotive context as well. The rise in prominence of autonomous driving and computer vision brings to the fore research in the area of the impact of image quality in camera perception for tasks such as recognition, localization and reconstruction. While the definition of “image quality” for computer vision may be ill-defined, what is clear is that the configuration of the image signal processing pipeline is the key factor in controlling the image quality for computer vision. This paper is partly review and partly positional with demonstration of several preliminary results promising for future research. As such, we give an overview of what is an Image Signal Processor (ISP) pipeline, describe some typical automotive computer vision problems, and give a brief introduction to the impact of image signal processing parameters on the performance of computer vision, via some empirical results. This paper provides a discussion on the merits of automatically tuning the ISP parameters using computer vision performance indicators as a cost metric, and thus bypassing the need to explicitly define what “image quality” means for computer vision. Due to lack of datasets for performing ISP tuning experiments, we apply proxy algorithms like sharpening before the vision algorithm processing. We performed these experiments with a classical algorithm namely AKAZE and a machine learning algorithm for pedestrian detection. We obtain encouraging results, such as an improvement of 14% accuracy for pedestrian detection by tuning sharpening technique parameters. We hope that this encourages creation of such datasets for more systematic evaluation of these topics.

## 1. Introduction

The fundamental concepts of image and video quality are well understood in consumer electronics, particularly in the multimedia context [1], and are the subject of standardization [2,3]. Image quality has been traditionally equated with “perceptual quality” and “naturalness,” or to how faithfully reproduction of the real world meets expectations of a viewer. Development of compression algorithms for multimedia content has driven many of the developments in defining and measuring perceptual image and video quality. In the automotive space, what “good quality” means is not so straightforward, with no single clear definition available [4,5]. This is compounded by the fact that video is necessary for two distinct applications: display to the driver (e.g., rear view and multi-camera surround view monitoring) and computer vision for advanced driver assistance systems. This is particularly pertinent in the move towards autonomous driving platforms, where camera systems are a diverse and key perception sensor that can provide structural, semantic and navigational information about the environment around the vehicle. As a result, computer vision algorithm performance, including deep learning, is expected to make significant jumps over previous systems. Originally, automotive surround view fisheye cameras were part of a viewing system to aid the driver. Such cameras are becoming very important for near field monitoring in automated driving applications [6]. Such distinct applications require different scene characteristics for optimal performance. The notion of what constitutes “good quality” for one does not necessarily equate to the same thing for the other. Consequently, although there is some basic work that has been suggested for monochromatic systems based on Johnson Criteria of Detection/Orientation/Recognition/Identification [7] and elaborated by [8] the established literature for specifically computer vision directed image quality is sparse relative to that for human vision image quality, which has been highlighted by e.g., [9,10]. This leads to a search for opportunities in the camera and processing systems for potential computer vision efficiency and performance gain.

Converting a raw signal from an image sensor to a viewable format includes various steps like demosaicing, denoising and gamma control, collectively referred to as Image Signal Processing (ISP). ISP is typically done by hardware engines either in the sensor itself, as a separate chip, or within a main System on Chip (SoC). Dedicated hardware is a necessity due to the vast level of processing required on the pipeline driven by the image resolution, bit depth, frame rate, number of exposures (HDR) and the number of processing steps. As an example, most colour image sensors employ the Bayer colour filter, and to obtain a usable/viewable image (e.g., full RGB or YUV), de-Bayering is necessary in a process called demosaicing. However, several of the ISP steps are designed to provide high degree of static visual performance to the end user for viewing applications, for example in traditional surround view applications. This may be unnecessary or even counter-productive for computer vision based applications.

Computer vision researchers, particularly in academia, typically use publicly available data for development, training and validation purposes, as bespoke data are difficult and expensive to obtain. Hardware setups would generally consist of off the shelf imaging systems with an on-board ISP that has little or no reconfigurability and a USB or Ethernet interface to capture YUV or RGB images. As a result raw pre-ISP image data is not always possible to capture. This generally forces researchers in computer vision to ignore the impact of ISP, through no fault of their own. However, it is likely that it has a very significant impact on computer vision algorithm performance. This is particularly critical for automated driving applications, where the performance of sensors and algorithms must be maximized.

In this paper, by providing a review of the areas of ISP, automotive computer vision and automated tuning, we aim to discuss the merits of the use of computer vision performance cost metrics to automatically tune the ISP parameters. By using ISP proxy algorithms, like sharpening, before the vision algorithm processing, we provide some evidence to support the position that this is an important topic. An additional goal, beyond explaining the importance of this topic, is to encourage the research community to create datasets for more systematic evaluation of these topics, such that all the details of the impact of each component of the ISP chain on computer vision can be investigated thoroughly. The rest of the paper is organized as follows. Section 2 provides a brief overview of ISP processing blocks, ISP tuning and computer vision algorithms. As they are cross-disciplinary topics, it will provide the necessary background for domain experts in one of these areas. Section 3 presents preliminary results and analysis, using both classical computer vision algorithms (AKAZE) and a machine learning algorithm for pedestrian detection. Section 4 discusses tuning algorithms and dual ISP pipeline, a hardware option that enables specific tuning of ISP for both computer vision and human viewing applications without conflict. Finally, the Section 5 summarizes and concludes the paper. This paper expands significantly on the authors’ previous conference paper [11].

## 2. Background

Here we provide some background on ISP Architectures and Computer Vision, with the aim to give the reader sufficient background to appreciate the remainder of the paper.

### 2.1. Related Work on ISP Impact and Tuning

Evolution of modern automotive machine vision systems did not follow a direct route. Essentially, automotive cameras took two distinct routes, one of providing an image to a user and another of providing machine vision input to an application such as an advanced driving assistance system (ADAS). OEM demands resulted in these two paths merging as they expect that both aspects can be provided by the same camera system. This has meant that the Key Performance Indicators (KPIs) of the distinct systems found their way into a common platform. This presents a number of challenges, as this combines visual image quality performance indicators, which are in the psychophysical domain, with traditional computer vision KPIs.

The role and development of visual KPIs in automotive has been elaborated elsewhere (e.g., [12]) but the semantics of visual perception do not easily lend themselves to simple elaboration, which led to the maxim “Image Quality should be FUN”, where FUN is an acronym for Fidelity, Utility and Naturalness, as these three categories are among the most frequently cited when engaging with a human user. Fidelity is generally represented by measuring the Modulation Transfer Function (MTF) of the imaging system, Utility by the ability to resolve objects of relevance to the viewer and Naturalness by the ability to provide visual representations of the world that are intuitive to the viewer.

Visual KPIs have evolved through a cycle of 3 distinct steps. Firstly, traditional component level metrics were applied to the signal of the respective components; essentially borrowing measurements from traditional optics and electronics. These included MTF for lens systems and signal-to-noise ratios for sensor signals. However, these only provided intuition about the quality of an image for extreme values (e.g., high MTF50 meant a very sharp image and low MTF50 meant a blurred image); they functioned well in component selection but not in interim visual assessment (e.g., [13,14]). A second approach was to create composite KPIs that are multivariate functions of simple features in input images. These borrowed from traditional feature detection or work on image compression metrics [15,16]. These included Universal Quality Index [17] and Structured Similarity (SSIM) [18], MS-SSIM [19], IFC [20], VIF [21], VSNR [22], FSIM [23] and salience weighted quality metrics [5]. “No-reference” techniques, which exclude the need for non-aberrant reference images when assessing the images are more desirable in real-time systems, but suffer from many of the same issues as reference techniques, which frustrate generalization of the interpretation of a metric’s measurement.

A third approach was to use visual psychophysical testing in the form of scientifically administered jury tests [24,25]. This arose for a number of reasons. The traditional component KPIs and multivariate functions did not sufficiently address the diversity of scenes or the trade-offs inherent in balancing ISP effects against each other, so it became again necessary for humans to view images and make judgments. In order to reduce or eliminate contributions from potentially confounding variables, classical visual psychophysical tests are deliberately set up to provide very constrained test environments [26,27] and attempts at more general automated visual psychophysical measurements are similarly highly artificially constrained and even then correlate poorly [28]. This, however, is the opposite of the general expectations of automotive imaging systems, which experience the greatest diversity of scene content of any imaging application, and automatic methods which leverage anticipated salience of objects to an observer in an automotive scene must be considered [5,29].

The sensitivity of computer vision algorithms to image quality KPIs was underlined recently “where performance drops catastrophically in response to barely perceptible changes” in automotive scenarios [30] as well as classification issues that can be induced by deliberately changing even single pixels [31,32].

The paper closest to the work we intend to do is [33]. The goal of this paper was to examine the role of the ISP pipeline in computer vision (traditional and CNN) to identify opportunities to reduce computation and save energy (to create a computer vision ISP mode). However, their tuning methodology is via disabling stages of the pipeline rather than tuning the parameters. They make some fairly important statements: (1) Most traditional ISP stages are unnecessary when targeting computer vision for their choice of algorithms. For all but one application they tested, only two stages had significant effects on vision accuracy namely demosaicing and gamma compression. (2) Their image sensor can reduce its bit width from 12 to 5 by replacing linear ADC quantization with logarithmic quantization while maintaining the same level of task performance. However, this work has a few limitations. They are doing black-box comparison of CV algorithms which are designed for ISP processed image. If the image is not ISP processed, the algorithms have to be adapted appropriately. For example, it is not optimal to run the same topological operators like SIFT [34] with and without demosaicing. The standard operator is not suited for a Bayer pattern image. Similarly for the absence of gamma compression, the operator could be adapted to handle it.

Recently, the paper [35] showed how to use simulation to understand the impact of different camera architectures. They analyzed the impact of the camera ISP on neural networks performance and their resilience to the variation of exposure. They compared the performance of 2 CNNs (SSD and RFCN) on detection. Each one was trained and tested with one of the following types of data: raw, linear or sRGB. Their results suggest that the training of a network with one type of image does not produce the same results for other camera settings. They have concluded that the best approach is to co-design the camera and the network.

### 2.2. Overview of ISP Architectures

**Figure 1:**
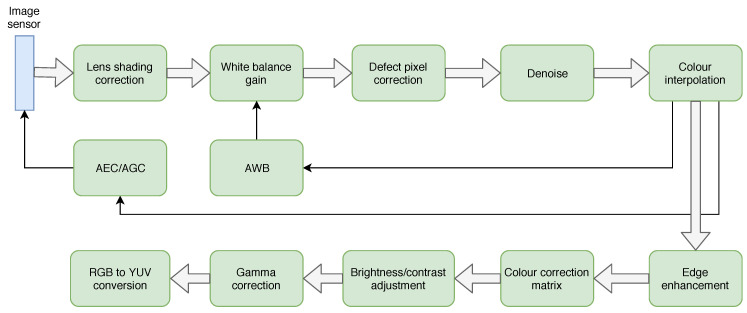
Representative ISP pipeline.

ISP is a processing block that converts a raw digital image into a usable image for a given application (typically a colour image for viewing). This conversion is quite complex and includes a number of discrete processing blocks that can be arranged in a different order depending on the ISP. An example image processing block is shown in Figure 1. Each ISP has its own unique features, but almost all ISPs have the same basic blocks and processing pipeline. The following is a brief description of each of the functional blocks identified in Figure 1 from the perspective of impact to computer vision algorithms.

**Lens shading correction**–corrects for brightness and color non-uniformity towards the image periphery. This is especially critical for fisheye lenses, where the light intensity fall-off, a property of lens transmittivity, can be significant at the image periphery. Lens shading correction is also required to correct color shading effects. As white light passes through the lens, the level of refraction varies, depending on the wavelength of light. A consequence of this is that, without correction, the center of a fisheye image typically has a reddish hue, and the periphery of the image has a cyan hue. If this is not corrected, there will be varying levels of color hue throughout the image. This will also impact the Auto White Balance (AWB) algorithm performance. Any detection algorithms which use color as an input would be more adversely affected. Lens shading correction is achieved by characterizing the lens optics and applying a spatially varying digital correction. This can have the side-effect of increasing noise at the image periphery which can impact machine vision performance [36].

**Auto white balance**–corrects for the colour temperature of the ambient lighting conditions, to preserve colour constancy (i.e., that a gray object appears gray regardless of the spectrum of light illuminating the scene) [37]. AWB statistics are calculated from the input image, and digital gains are applied to the red, green and blue colour channels to correct colour casts due to ambient lighting. Accurate AWB is critical for any machine vision algorithm that uses colour as an input. Lane marking detection would be particularly vulnerable to AWB inaccuracy. For example, distinguishing between a yellow and white lane marking under sodium vapour street lighting is a very challenging use case. Other algorithms, including traffic sign and traffic light recognition would also be affected by AWB performance. AWB is an example where there may be a trade-off between image Naturalness and Utility. One use case is night scenes illuminated by sodium vapour lighting. There are a number of variants of sodium vapour lighting, but all have quite low colour temperatures and appear orange or reddish to a human observer. Typically, for human viewing applications, it is desirable to tune the AWB response of the camera to match the expectations of a human observer as closely as possible. However, this may not be necessary for machine vision, or may even negatively affect machine vision performance. An alternative approach to white balance for machine vision may be to correct for sodium vapour lighting, making the image “appear neutral”, as if illuminated by a D65 or similar source. In this case, the image may appear unnatural, but salient objects, including yellow road markings may be more salient. It may even reduce training set requirements i.e., it may no longer be necessary to train algorithms to detect road markings in sodium vapour scenes. As far as the authors are aware, there are no studies which have examined this question in detail.

**AEC/AGC**–Auto Exposure Control [38], Auto Gain Control [39]. AEC/AGC block controls the exposure and gain of the image sensor. The exposure and gains for the next captured frame are calculated based on the weighted average of the current exposure. This is the only true feedback loop within the ISP. Accurate AEC/AGC performance is crucial for machine vision performance. An underexposed image will have poor SNR and contrast separation, and an overexposed image will have information loss in the scene highlights. Also, depending on the application, different exposure weighting schemes may be considered. For example, for a headlamp detection algorithm, it may be acceptable to underexpose the image, to ensure the headlamps do not bloom. Conversely, if detection of objects in shadows is the primary concern, it may be acceptable to overexpose large sections of the image, to ensure details in shadows are captured. The goal of HDR imaging is to ensure both highlights and shadows are captured simultaneously. Motion blur is another consideration given the automotive application. Depending on the perception task, it may be acceptable to underexpose the image and boost the brightness level digitally, to avoid motion blur artifacts.

**Defect pixel correction**–corrects for defective pixels on the image sensor. The number of defect pixels in an image sensor increases over the lifetime of the sensor. Defect pixel correction algorithms are the reason they are typically not visible in digital images. Defect pixels have the potential to impact machine vision algorithm performance. Su et al. [31], showed that with just a single pixel adversarial perturbation, 70.97% of the natural scenes tested could be perturbed to at least one target class with a 97.47% confidence on average. This admittedly extreme example demonstrates the potential for defect pixels to impact machine vision performance, among similar vulnerabilities which have been elaborated [40].

**Denoise**–reduces the appearance of noise in the image [41]. This is typically achieved through the use of 2D noise filtering. In most ISPs, there is a trade-off between noise removal and texture preservation. Excessive denoising may significantly improve SNR, but at the cost of high frequency information. 2D low pass filtering is a preprocessing step in many computer vision algorithm pipelines but excessive denoising resulting in the removal of valid high frequency data can impact detection of image gradients. Image gradients, which are key requirement for feature detectors, line detection and optical flow among other operations, are a vital component of the pipeline of the majority of computer vision algorithms.

**Colour interpolation**–converts a sensor’s raw colour data, typically captured using a Bayer Colour Filter Array (CFA) into a colour RGB image. This process is also known as demosaicing [42]. Demosaicing is one of the most critical operations of any ISP. Modulation Transfer Function (MTF) and image noise are directly affected by demosaicing. A number of image artifacts can also be introduced by demosaicing. Examples include zippering/staircase artifacts on edges, and aliasing/false colours in high frequency patterns. Many of these effects can be mitigated by more complex demosaicing filters. There is typically a trade-off between computational load and image quality. The introduction of noise, in particular horizontal and vertical edged noise, can result in incorrect feature extraction which is typically based on strong vertical and horizontal image gradients. Repeated edge effects such as the staircase artifacts can cause incorrect feature extraction and matched as well as producing incorrect optical flow along the edge rather than in the direction of motion.

**Edge enhancement**–processing block which enhances edges, normally to make an image appear sharper to a human observer. However, excessive edge enhancement can introduce artifacts such as halos around high contrast edges, and can also accentuate noise. Excessive edge enhancement can negatively impact gradient based algorithms through enhancing noise and by the artificial creation of duplicate edges on the overshoot and undershoot of a sharpened edge.

**Colour correction matrix**–corrects for cross-talk between adjacent sensor pixels [43]. Crosstalk is a pixel level phenomenon where colour information from one pixel contaminates an adjacent pixel. It can be both optical and electrical in nature. Colour correction is required to correct for crosstalk related colour inaccuracies. In some cases, colour correction can also introduce or exacerbate colour noise. This will occur when there is a significant mismatch between colour hues, which requires high levels of digital correction. Increased noise as well as colour inaccuracy has the potential to negatively impact machine vision performance.

**Brightness/contrast adjustment**–the implementation details of this block vary significantly, but the key goal is to enhance image contrast and digitally adjust image brightness. Typical contrast enhancement algorithms would include histogram stretch, histogram equalization, local and global contrast adjustment algorithms (e.g., CLAHE) etc., Contrast enhancement can improve contrast separation between different grey levels. This can be advantageous for machine vision performance. However, excessive contrast enhancement can accentuate noise and reduce SNR which can negatively impact computer vision performance. Brightness and contrast tunings for human and machine vision purposes can be mutually antagonistic.

**Gamma**–gamma correction block adjusts the contrast associated with differently light levels differently, to increase the salience of features. Gamma correction is crucial for viewing applications. Without it, higher image bit depth would be required to avoid visible posterization effects. For machine vision applications, its impact is less clear. The contrast of shadow details is accentuated, but contrast in highlights is compressed. This can potentially adversely affect traffic sign recognition or headlamp detection algorithms, for example.

### 2.3. Computer Vision Algorithms for Automotive Applications

#### 2.3.1. Classical Computer Vision

When we refer to classical computer vision (CV) we are referring to the process of automating tasks that the human visual system can in general perform without usage of deep learning approaches. Deep learning (covered in the next section) can be considered as a sub field within modern computer vision as it is quickly becoming the state of the art for almost all CV tasks.

In the case of automated driving many sub-domains of computer vision are utilized to extract information about the environment around the vehicle including reconstruction, object recognition, 3D pose estimation, machine learning and motion estimation [44]. The following section briefly describes two traditional computer vision techniques, 3D reconstruction and road marking detection, that are regularly utilized in automated driving functions. These are examples of CV being utilized for automated driving functions to give context to the importance of robust and accurate computer vision output and by association the importance of the image that is processed. A more detailed survey of computer vision algorithms for automotive applications is presented in [45].

**Figure 2:**
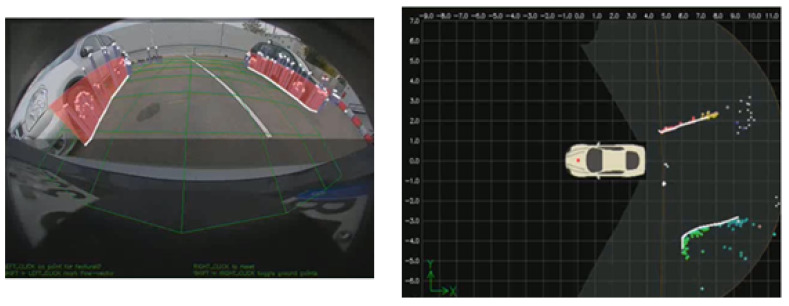
Reprojected and top view of 3D reconstruction.

**3D reconstruction**–3D reconstruction refers to the set of algorithms aimed at obtaining a representation of the spatial structure of the environment within the sensor’s FOV. In the context of automated driving, it is the primary mechanism by which computer vision can be used to create a metric map of the environment around the vehicle. There are two main types of depth perception techniques for cameras: namely stereo and monocular [46], with the primary advantage of stereo cameras over monocular systems is the ability to sense depth even without camera motion, whereas monocular is attractive as it a lower cost option. Stereo vision works by solving the correspondence problem for each pixel, allowing for disparity mapping of pixel locations from the left camera image to the right camera image. The distances are proportional to the physical distance of the corresponding world point from the camera. Using the known camera calibrations and baseline, the 3D position in the world for each pixel can be resolved. Figure 2 shows an example of sparse 3D reconstruction.

Monocular systems also have the ability to sense depth, however, camera motion is required to create the baseline for reconstruction of the scene. This method of scene reconstruction is referred to as structure from motion (SFM). Pixels in the image are tracked or matched from one frame to the next using either sparse or dense optical flow or feature extraction and matching techniques. This is main step which happens on the image domain and it is commonly accomplished by feature matching algorithms like SIFT, AKAZE, etc., [47] which will be one of the main algorithms we evaluate for impact on ISP. The calculated motion of the camera between the processed frames as well as the camera calibration, are used to project and triangulate the world positions of the point correspondences. Bundle adjustment is a commonly used approach to simultaneously refine the 3D positions estimated in the scene and the relative motion of the camera, according to an optimality criterion, involving the corresponding image projections of all points. Monocular depth has been the subject of text books for quite some time [46].

**Figure 3:**
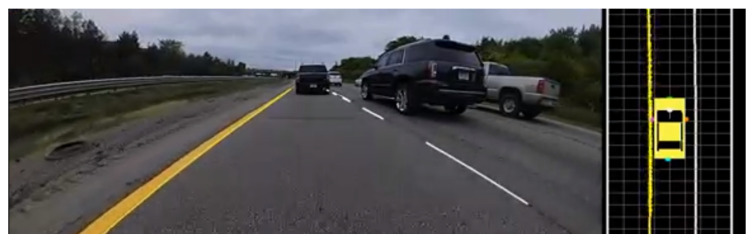
Example of road marking detection.

**Figure 4:**
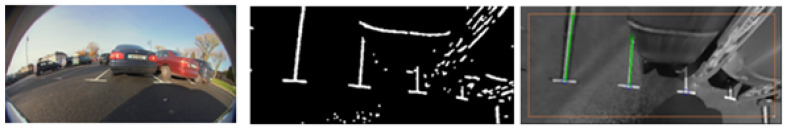
Example of parking slot marking recognition.

**Road marking detection**–in automated driving, the detection of road markings is naturally a critical component of any sensing system. The detection of lane boundaries (example Figure 3) is well understood in the automotive computer vision industry, being among the first automotive computer vision products created, however, it remains the source of active research given the recent context of automated driving [48,49]. Perhaps a little less obvious, but equally important, is the detection of parking marking for automated parking systems. In vision, lane marking detection can be achieved using image top-view rectification, edge extraction and Hough space analysis to detect markings and marking pairs [50]. Figure 4 gives an example of the results from a similar approach, captured using 190° horizontal field of view parking camera [51]. The same authors also propose a different approach based on the input of a manually determined seed point, subsequently applying structural analysis techniques to extract the parking slot [52]. Alternatively, a pre-trained model-based method based on HOG (Histogram of Oriented Gradients) and LBP (Local Binary Patterns) features, with linear SVM (Support Vector Machine) applied to construct the classification models is proposed in [53]. Regardless of the specific approach taken, what is clear is that the detection of road markings is necessary for a complete automated driving system, from highway driving to parking.

#### 2.3.2. Deep Learning

In the last 5 years state of the art computer vision techniques have rapidly evolved, with deep learning and specifically convolutional neural networks (CNN) at the heart of this. CNNs have enabled a large increase in accuracy of object detection leading to better perception for automated driving [54]. It has also enabled dense pixel classification via semantic segmentation which was not feasible before [55,56]. Additionally there is a strong trend of CNNs achieving state-of-the-art results for geometric vision algorithms such as optical flow [57], moving object detection [58], structure from motion [59], re-localisation [60], soiling detection [61] and joint multi-task models [62]. The rapid progress in CNN has led hardware manufacturers to include a custom hardware to provide a high throughput of over 10 Tera operations per second (TOPS). Additionally the next generation hardware will have dense optical flow and stereo hardware accelerators to enable generic detection of moving and static objects.

**Figure 5:**
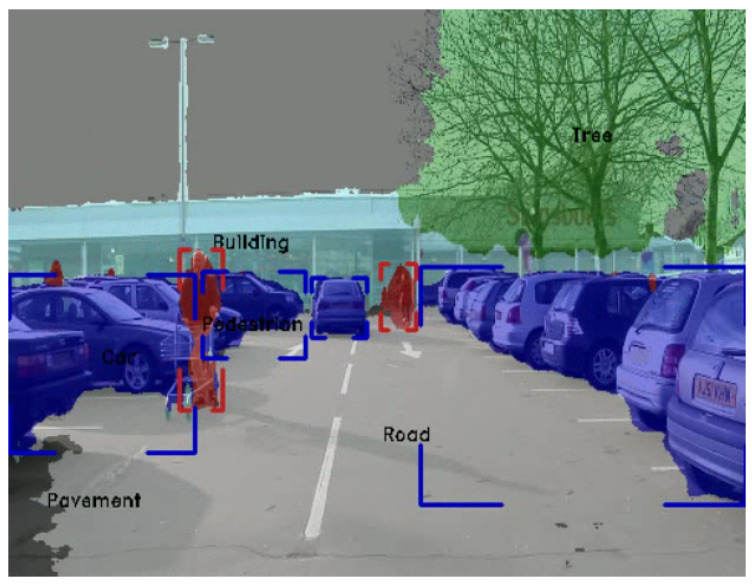
Semantic Segmentation of a typical automotive scene.

Semantic image segmentation has witnessed tremendous progress recently with deep learning. Semantic segmentation is targeted towards partitioning the image into meaningful parts with various applications utilising this information. It has been used in robotics [63,64,65,66], medical applications [67,68], augmented reality [69], and most prominently automated driving [70]. Figure 5 illustrates an example of the semantic segmentation output in an automated driving setting. There were mainly three subcategories of the work that was developed. The first [71,72,73] used patch-wise training to yield the final classification. The second subcategory [74,75,76] was focused on end-to-end learning of pixel-wise classification. It started with the work in [74] that developed fully convolutional networks. Finally, the work in [71,75,77,78,79,80] focused on multiscale semantic segmentation.

**Figure 6:**
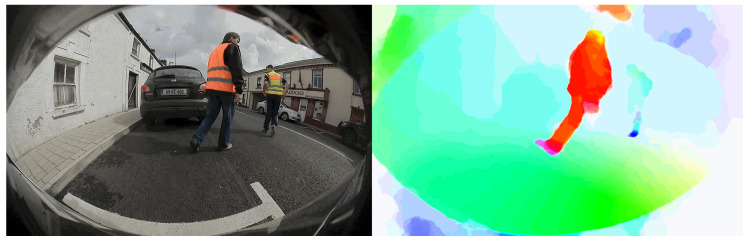
Illustration of geometric deep learning algorithm for computation of dense optical flow.

As discussed, geometric computer vision tasks are an essential part of Autonomous Driving (AD) systems. Specifically, we refer to multi-view geometry algorithms which estimates relative motion and depth from multiple images. Visual perception for autonomous driving is highly influenced by the accuracy of these geometric applications like optical flow [81], structure from motion [82], visual odometry, simultaneous localization and mapping (SLAM) [60]. These algorithms are deeply investigated using the classical approach for decades within the computer vision community. However, there is an emergence of deep learning based approaches for these algorithms. Deep learning has played a significant role in object detection and segmentation and has become a mature solution for autonomous driving. Recently, deep learning has become the state of the art method for certain tasks like flow and depth estimation just by using convolutional neural network (CNN) models without incorporating geometric structure. There are also attempts at using CNN for visual SLAM, visual odometry and calibration. Motion estimation covers both dense optical flow estimation and moving object segmentation. Figure 6 illustrates a geometric deep learning algorithm for computation of dense optical flow. Depth estimation is a key algorithm in AD for localizing recognized objects around the car relative to the vehicle and we study supervised, unsupervised or semi-supervised approaches. In principle, the CNN learning algorithm should be able to learn the necessary transformations which are optimal for the algorithm KPIs. However in practice, there is a lot of empirical evidence to incorporate known transformations as inductive bias for better performance [83].

### 2.4. Discussion

Computer vision plays a very significant role in vehicle automation providing an abundance of environmental information for the vehicle to make vital assistance and more importantly safety critical decisions. It is clear for both traditional and deep learning based functions that the formation of the image itself, being the only raw sensor input into these functions, is vital to achieve the robustness, availability and accuracy requirements to achieve higher automation levels. The push towards higher levels of vehicle automation is driving the performance requirements of all CV functions. The pixel level processing stage of both traditional and deep learning based CV functions is reliant on the fidelity of the image input. Independent of the approach used to extract feature level data from the image, be it traditional feature extraction or the encoder of an encoder-decoder neural network, the features such as points, edges, corners, blobs or ridges extracted are only dependable if the image provided accurately represents the scene being imaged. This accuracy of image production, as stated previously, can be at odds with perceived visual quality of a human observer. The ideal image representation for CV is one that provides sufficient, consistent and repeatable contrast for all scene structure (geometric, texture, colour, reflectivity, etc.) which is independent of lighting, temperature, environmental conditions and the scene structure, however, this is not achievable due to the vast number of system and scene variables either not measurable, difficult to model or not accurately reproducible on today’s imaging hardware. Some of the important image characteristics that impact feature extraction at a pixel level include image compression, blur/sharpening, contrast, noise, colour compression and a selection of these are investigated in this study. While all these image characteristics are not controlled in their entirety by the ISP it does however heavily influence their presence, weighting and impact in the produced image. While recent studies have investigated the impact of some of these image characteristics on CV performance [84,85] and others have proposed the possibility of ISP tuning or adaption for mobile applications [33] very few if any have researched the impact and implications for automotive applications.

## 3. Empirical Analysis of Image Processing Parameters’ Impact on Computer Vision Algorithms

**Figure 7:**
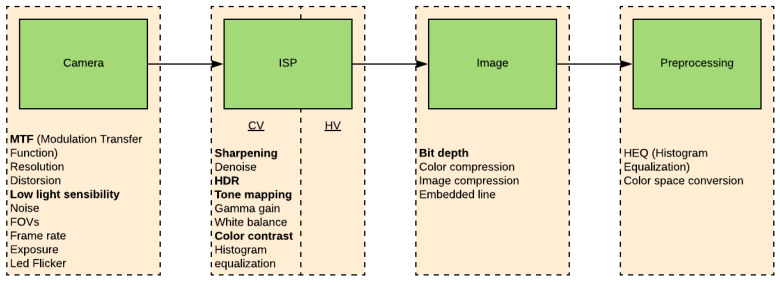
Pipeline overview. Key ISP modules which impact CV performance are marked in bold.

In typical advanced driving assistance systems (ADAS) or automated driving applications that make usage of surround high field of view cameras, a single ISP is shared for Human Vision (HV) and Computer Vision (CV) functions. The signal processing of the raw image created is primarily driven by human visual quality of the surround view system. The tuning of an ISP pipeline after development of computer vision or deep learning applications is likely to have an adverse impact on algorithm performances as algorithm performance is not part of the optimization loop. All algorithms could be impacted by for example SNR degradation introduced by changes. Geometric vision algorithms are inherently sensitive to ISP pre-processing changes as the pixel level operations such as feature extraction generally rely on fixed statically tuned kernel sizes and parameters as well as fixed saliency thresholds. Regarding deep learning algorithms, they would be the more robust as long as their models are trained with a high variability of training samples. For example in the paper [84], the authors observed a reduction of performance of different Deep Neural Network architectures under blur and noise, while being resilient to contrast and JPEG compression. With the push for visual perception improvements to aid autonomy and the release of SOCs with integrated ISPs, dual ISP pipelines for both human vision and computer vision is now achievable. As explained earlier, raw images are generated by the image sensor. To be viewable by devices, the raw data has to be processed by an ISP (Figure 7).

### 3.1. Overall Methodology and Test Settings

#### 3.1.1. Test Setup

**Figure 8:**
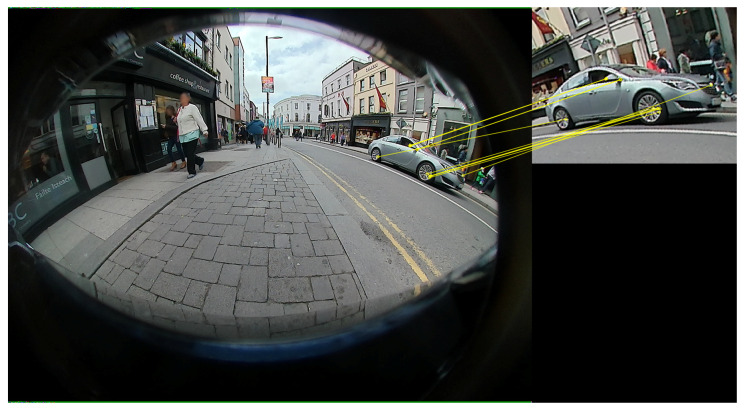
Illustration of ORB based feature matching.

This paper gathers and expands results presented in the authors’ previous publications [11,86]. It principally focuses on the results obtained for sharpening and contrast from a pixel-level processing perspective and shows preliminary results of impacts on KPIs for a light pedestrian detection (PD) algorithm pipeline consisting of an Adaboost candidate generation followed by a small CNN-based candidate validation. Sharpening and contrast are typical ISP processing blocks that more than any other parameters, are driven by subjective experience rather than objective fitness for the application. As ISP tuning for computer vision is currently an overlooked topic, no dataset of raw images with vision KPIs is available. The data used in this study have been recorded internally using fisheye cameras mounted in a car. The videos/images have been recorded in the street in driving scenario or parking situations and contain objects, such as pedestrians, cars, traffic and road signs, lines, etc., Note that these fisheye images are not raw and therefore have basic ISP applied prior to test. Because of this lack of pre-ISP images, we could only do minimal proxy tests to ”mimic” some block of ISP (sharpening and contrast). These tests will be rerun in the future with raw images by tuning an ISP. The pixel level study compares original and processed images on: edge detection (using Sobel filter) [87], binarization and closing (morphological operation) [88]. By looking for inliers on the 100 most salient matched points (according to their distance), the impact on features extraction has been studied. The homography between two images is computed using RANSAC. The study looks at the effect of ISP on each feature extractor independently. These pixel-level study has been run over 100 images. In computer vision, a feature is a salient part of an image (point, blob, edge etc.) which reduces the amount of data to be processed, focuses on the relevant parts of the image, maybe temporally robust and is further processed by the next stage of the CV algorithm pipeline. The feature descriptors/detectors used in this investigation are invariant to uniform scaling, orientation and illumination [47]: Scale-invariant feature transform (SIFT) [34], Speeded up robust features (SURF) [89], Oriented FAST and Rotated BRIEF (ORB) [90] and Accelerated-KAZE (AKAZE) [91,92]. The features are extracted in six frames in total (n…n+5), and matched between frame *n* and each of the successive frames in the sequence (n+1 to n+5) (Figure 8). The previous algorithms (edge detection, binarization, closing and feature detectors/descriptors) have been chosen as they are largely used in computer vision and deep learning algorithms.

For the analysis of the impact on key performance indicators (KPIs), a catalog of 20 videos has been used for testing. A typical pedestrian detection (PD) algorithm have been performed on all videos. The algorithm detects pedestrians up to 8 m depending on the light level, pose, contrast, etc., and draws bounding boxes around the detected pedestrian. The metric used to quantify the impact is a standard KPI used in the industry. Our KPI metric is intersection over union (IOU) between the annotated and the detected bounding boxes. Larger IOU indicates better accuracy and true positive is defined when IOU is above a threshold for a specific object instance.

#### 3.1.2. Sharpening

**Why is sharpening used in viewing applications?** The human vision system is highly sensitive to edges and fine details in images and is adept at adapting to distinguish lines of contrast. Edges and details are principally located on high-frequencies. However, a camera is composed of a finite number of pixels, which means that only a finite frequency of data can be adequately captured and represented in an image. In images, borders between objects are perceived because of the intensity change (higher intensity transitions equates to a sharper image). The intensity transitions between adjacent pixels are related to the derivatives of the image (spatial differentiation). Sharpening is widely used to post process blurred images by enhancing the scale of the intensity transitions. Increasing the difference between dark and bright regions accentuates edges. Sharpening can be potentially beneficial in wide FOV lens images as it has compensation for the OTF (Optical Transfer Function). A decrease in resolution towards the periphery of the images occurs in this case which can be enhanced by sharpening.

**Techniques used:** Two techniques were used to sharpen images. Both techniques were applied with different parameters. The first technique is using Laplacian filters to enhance fine details contained in the high-frequency regions. The kernels are designed to increase the brightness of the center pixel relative to the original ones. The Laplacian operators ($L_{4}$ and $L_{8}$) are a 2D isometric measure of the second spatial derivative of an image:

$$L_{4}=\left(\begin{array}{ccc} 0 & -1 & 0\\ -1 & 4 & -1\\ 0 & -1 & 0 \end{array}\right),L_{8}=\left(\begin{array}{ccc} -1 & -1 & -1\\ -1 & 8 & -1\\ -1 & -1 & -1 \end{array}\right)$$

**Figure 9:**
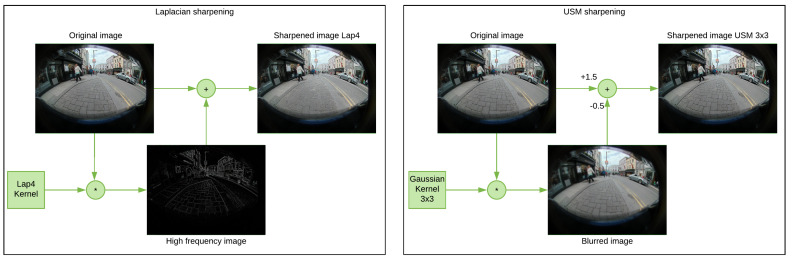
Sharpening methods | Left: using Laplacian filters/Right: using USM.

The second technique used is the unsharp masking (USM). It uses an unsharpened (blurred) negative image mask of the original image, combined through a per-pixel weighted sum with the positive original image, to create a sharpened version. Here we use a set of Gaussian kernels with varying sizes (3×3, 9×9 and 19×19) to create the blurred image, and use a weighting of 0.5 on the blurred image. The Figure 9 (left: Laplacian, right: USM) shows how the filters have been applied.

**Figure 10:**
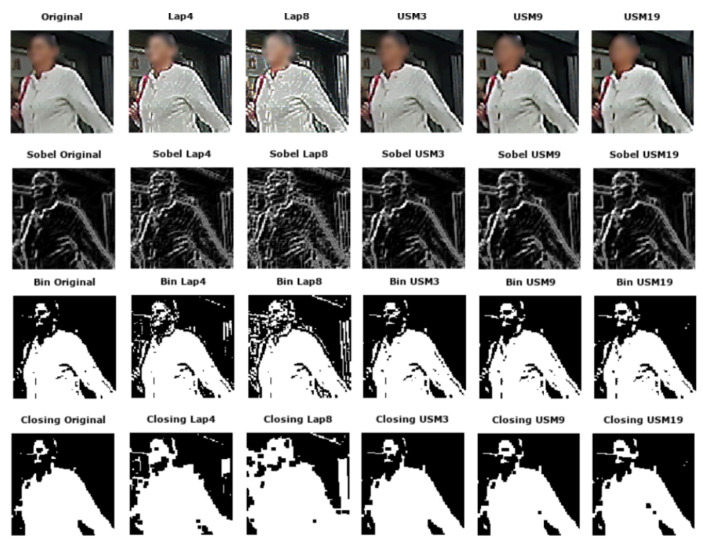
Samples of sharpened images with various parameterizations.

**Visual Analysis:** The two techniques have been applied to the images. All outputs look sharper and the acutance is enhanced as the original blur appears to be removed. From a visual perspective, it is observed that images sharpened with Laplacian filters are noisier than images sharpened with USM. Halo artifacts appear after each treatment but are more noticeable in the Laplacian images. Regarding edge detection, the noise in the images sharpened with the Laplacian operator is detected as edges in some cases contrary to the halo artifacts created by USM as they are not strong enough to be detected as edges. The results obtained by the binarization are similar to the previous observations as there are no big differences between the original binarized and the sharpened with USM binarized images. The noise of images sharpened with Laplacian filters is noticeable. As the morphological closing operation is performed on binarized images, similar conclusions are made. (Visual differences: Figure 10).

**Figure 11:**
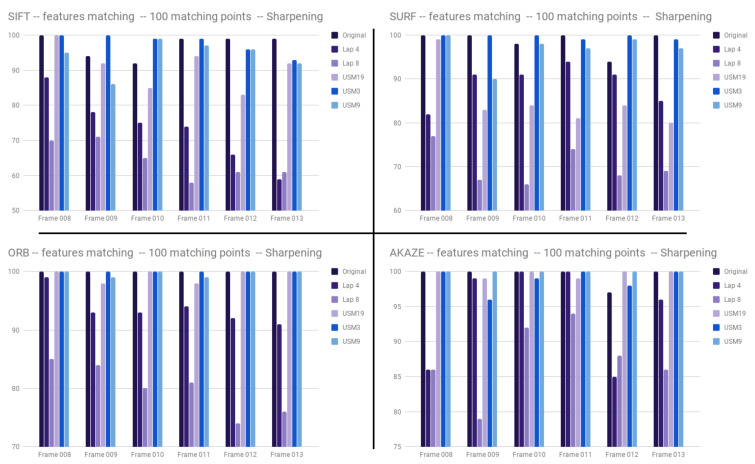
Inliers percentage depending on the frame and the sharpening technique for SIFT, SURF, ORB and AKAZE.

By observing results given by the feature detectors, it can be seen that sharpening has an impact on feature detection. Matching the original image with images created by Laplacian filtering always returns a lower percentage of inliers. The results of our tests show that the USM method can have a positive or negative impact on performance (Figure 11).

**Figure 12:**
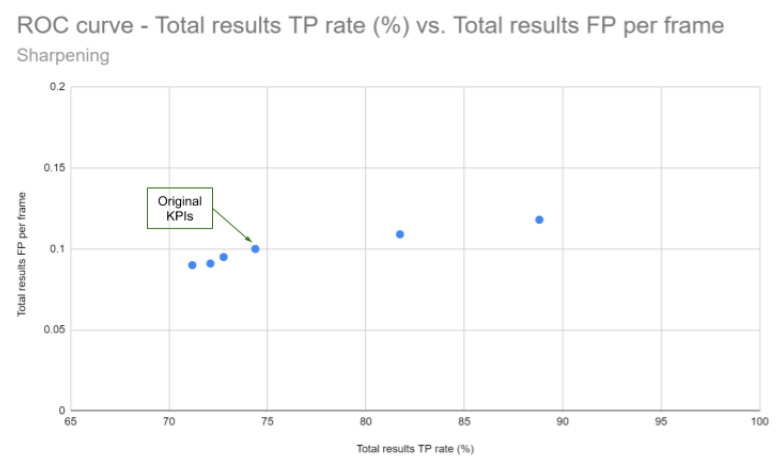
ROC curve showing TP rate vs. FP per frame for the 5 configurations tested and test on original images.

**Figure 13:**
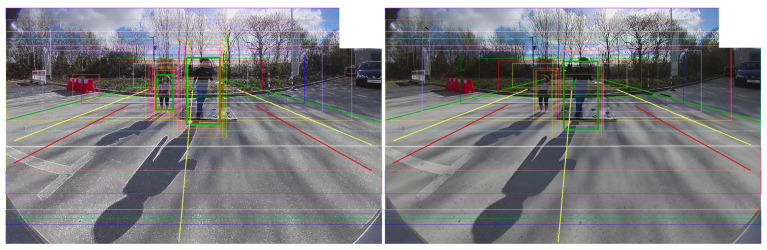
Image after sharpening: (Left: Lap8 & Right: USM9). When the PD algorithm detects a pedestrian, a green bounding box is drawn (all other lines are for debugging).

**Quantitative Analysis:** As explained earlier, the KPI values have been calculated for a catalog of 20 videos. The Table 1 and the ROC curve in Figure 12 show the results obtained for the two sharpening techniques. By comparing the KPI values between the unprocessed and the sharpened videos, it can be observed that the Laplacian filters improve the KPI values: +14.43% (Lap8) and +7.35% (Lap4) by keeping the FP per frame around the same value as the original test. The three configuration used for the unsharp masking give the opposite results (lower TP percentages). Visually, the unsharp masking technique gives better images if it is compared with the laplacian filtering where a lot of noise and halo artifacts appear. This highlights that what is visually appealing for human vision is not necessarily good for computer vision algorithms (Figure 13).

#### 3.1.3. Contrast

**Why is Contrast used in viewing applications?** In visual perception of the real world, the contrast is determined by the difference between the brightness and colour of objects within the same field of view. The human visual system is capable of perceiving the world similarly regardless of illumination changes because it is more sensitive to contrast variation rather than absolute luminance. Contrast enhancement is an important tool in photography as it is used to create eye-catching images and to direct viewers’ attention to an object.

**Techniques used:** Two techniques were used to contrast images namely Histogram Equalization (HE) and Contrast Limited Adaptive Histogram Equalization (CLAHE) [93]. The histogram equalization technique impacts the global contrast of the image and distributes the intensities equally and is useful for images having bright and dark areas in both background and foreground. The CLAHE technique is an improvement of the AHE (Adaptive Histogram Equalization) technique which improves the contrast of an image. Several histograms are computed for each section of the image where the intensity values are redistributed.

Results:

**Visual Analysis:** It can be observed that the two techniques tested give different outputs. The histogram equalization results in images shifted to a different intensity. The consequence is that some high intensity details (such as clouds, some boundaries, etc.) disappear from the image and others less intense areas become more distinguishable.

**Figure 14:**
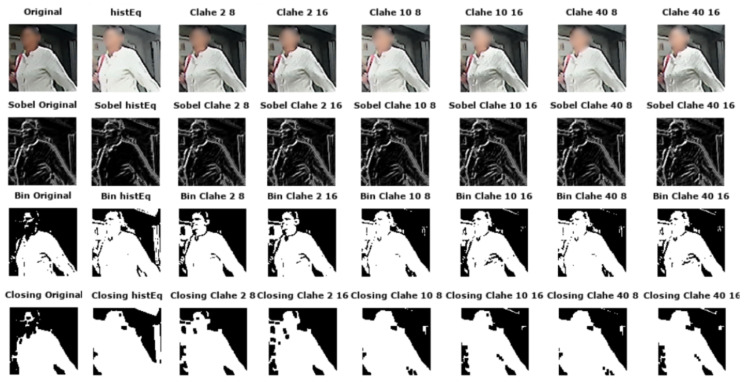
Samples of contrast enhanced images with various parameterizations.

As CLAHE is an adaptive method, the images created show that the dark areas from the original image become darker and the light become lighter. Images appear to contain more details. Halo artifacts are created along edges and the noise increases with the size of the tiles and the clip limit applied. It appears that the noise is proportional to the clip limit size (bigger clip size implies more noise in the image). By observing images after edge detection, it can be observed that Sobel images of the histogram equalized images are very similar to the original whereas noise is detected as edges in CLAHE. Differences between original and processed images are highlighted after binarization. The HE image has lost a lot of information. However, as the image is brighter, it is now possible to distinguish and recognize features that were not visible on the source image. Halo artifacts created by the CLAHE method are visible on binarized images. The closing images are affected differently depending on the contrast technique used. There is a big loss of information in the histogram equalized images as the image is brighter in all areas. The CLAHE images give more details when the clip limit and the size of tiles are bigger (Figure 14).

**Figure 15:**
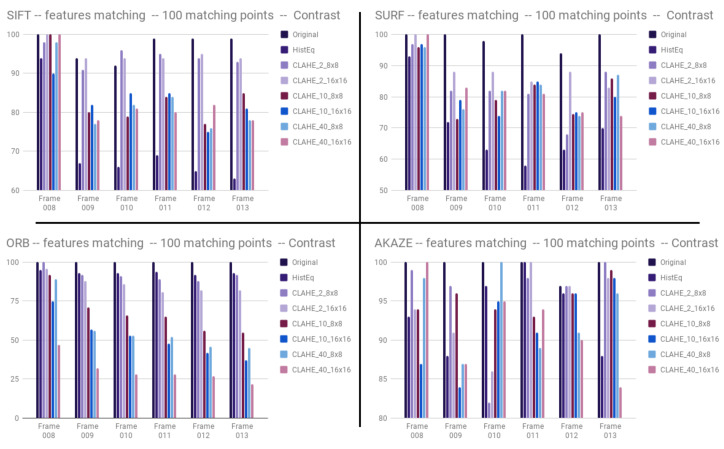
Inliers percentage depending on frame number and contrast method using. SIFT, SURF, ORB and AKAZE.

Feature matching tests gave similar results for the sharpening. Figure 15 shows that contrast has a negative impact most of the time on the different features detectors. Only the frame 10 with SIFT gives higher percentages of inliers after contrast. The other conclusion we can get from this figure is that some percentages of inliers in the AKAZE test after contrast are equal to the original percentages of inliers (depending on the parameters of the contrast algorithm, the impact can be negative or none). These preliminary results on a small set of frames show that contrast enhancement does not positively impact computer vision as it does for human vision. Experiments have to be repeated on larger datasets having raw annotated data which is not currently available. The inlier percentages drop in most cases when testing feature matching between the non-processed original image and the processed one. The Figure 15 shows the results obtained.

**Figure 16:**
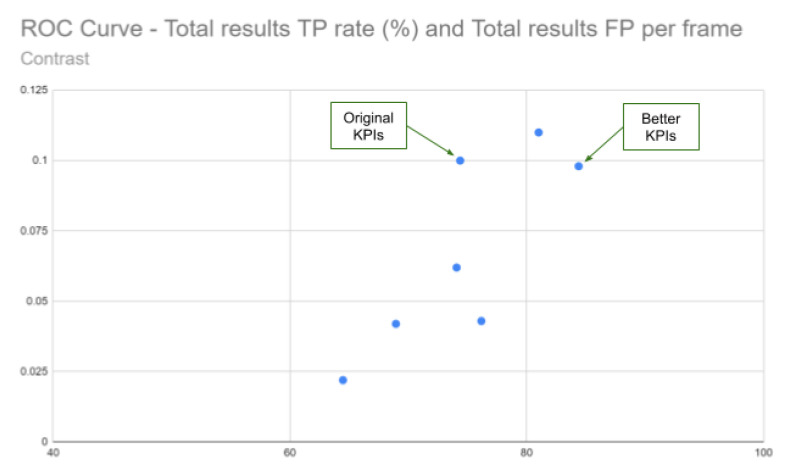
ROC curve showing TP rate vs. FP per frame for the 6 configurations tested and test on original images.

**Quantitative Analysis:** The Table 2 and the ROC curve in Figure 16 show that a contrast modification can have a real impact on KPI values. This impact can be positive or negative on CV performance. The CLAHE tests done using 3 parameters sets give higher TP percentages: +10.01% (CLAHE_2_16), +6.63% (CLAHE_2_8) and +1.8% (CLAHE_10_16). Regarding FP per frame, it can be observed that CLAHE_2_8 and CLAHE_2_16 are very similar as the original test and way better in CLAHE_10_16 (the lower the FP per frame is, the better).

### 3.2. Sharpening and Contrast’s Filters Tuning

This section presents the results presented at the Electronic Imaging conference [11]. In this experiment, a catalog of 93 videos have been used for testing and both sharpening and contrast have been applied before the pedestrian detection (PD) algorithm. To get ground truth results, the pedestrian detection algorithm has been run for the entire catalog. To limit the configuration space, one filter per techniques have been tested: Laplacian filter for sharpening, which has 1 parameter that has two possibilities and CLAHE technique for contrast that has two parameters: clipLimit: [1,15] and tileSize: 8×8 or 16×16. The objective here was to optimize the PD KPIs: maximizing the TP rate while maintaining the FP rate as low as possible. To achieve this objective, we propose to calculate a *compromise* value (Γ) in order to jointly optimize TP rate ($r_{TP}$) and FP rate ($r_{FP}$) values:

$$\Gamma =r_{FP}\left(1-r_{FP}\right)$$

**Figure 17:**
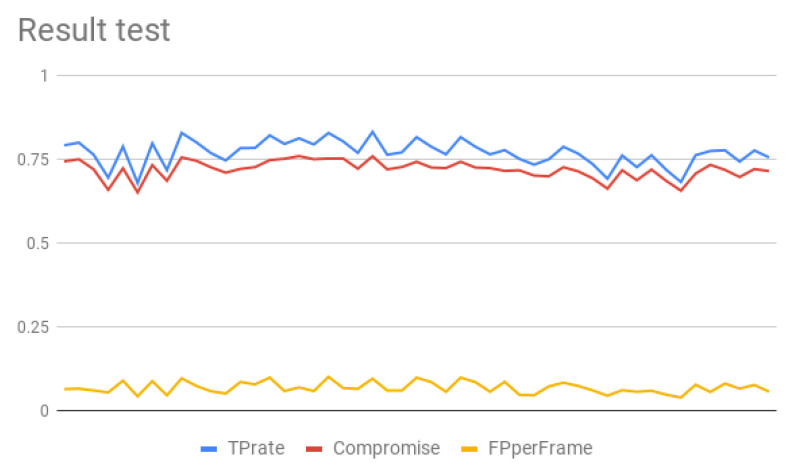
TP rate (%) vs. FP per frame for contrast.

The graph of the Figure 17 shows the three metrics per configuration (TP rate, FP per frame and compromise values). It can be observe that the TP rate value vary from 0.68 up to 0.83. The FP per frame also varies significantly, from 0.039 to 0.101.

In Table 3, we establish the best configurations by TP rate and compromise value.

The best TP found in the configuration is 0.83, which is about 0.045 increase over the original. By looking at the FP per frame value, it can be observed that it is almost doubled (0.095) compared to the original. However, the compromise value takes in account the TP rate and the FP per frame, and is perhaps a better measure. The last column shows the KPIs values if sorted by compromise value, the best TP rate is 0.81, which is more that 0.025. In this case, the FP per frame is still low (0.069).

**Figure 18:**
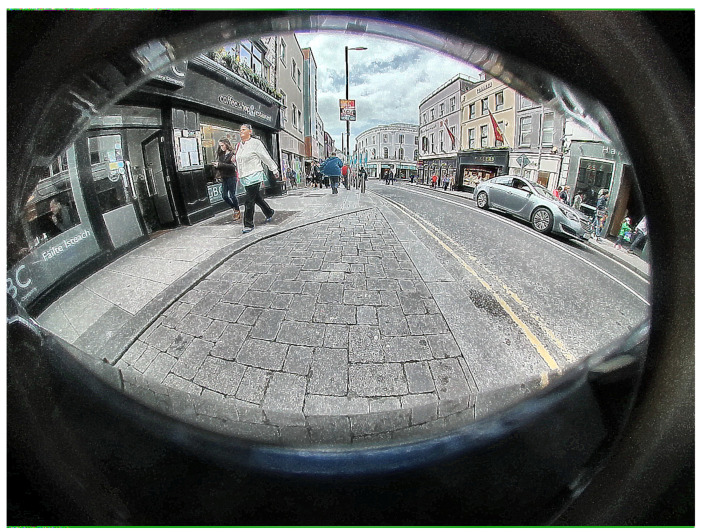
Image sharpened and contrasted using the parameters given by the best configuration found when sorted by compromise value (Lap8, clipLimit = 2 and tileSize = 8 × 8).

Table 4 shows the parameters value of the best configuration found when the sort has been made per TP rate or per compromise value. The Figure 18 shows an image processed by the parameters value given by the best configuration found when the configuration are sorted by compromise value. The “image quality” of this image is far to be considered as a ”good looking” image if we consider human vision applications.

### 3.3. Discussion

The results obtained in this pixel level study has shown that there are visual and measurable impacts on computer vision algorithms performance implied by the modification of two blocks of the ISP pipeline. Firstly, the visual differences are observed after the application of the Sobel operator (edge detection), the binarization and the morphological operation. The effect on the performance of feature extraction (such as SIFT, SURF, ORB and AKAZE) has been highlighted by the calculation of percentages of inliers for each method.

The tests on pedestrian detection algorithm KPI show the important improvement or regression of the algorithm performances just by using post-processing filters. In the experiment where filters have been applied one by one, it could be observed that TP percentages between original and processed image vary from +14.43% to −3.2% (sharpening) and from +10% to −10% (contrast).

In the last experiment made on a bigger video catalog, the results confirmed the conclusion drawn before: ”image quality” has a measurable and significant impact on computer vision. By tuning the two filters we could improve the TP rate by maintaining the FP rate at a low level. It is a strong indicator that ISP tuning for computer vision is a significant potential area of investigation in order to obtain the optimal performance of computer vision algorithms, both traditional feature extraction and machine learning algorithms.

## 4. Future Work: Specialized ISP for Computer Vision

### 4.1. Tuning Algorithms

Tuning algorithms typically requires a scalar metric which can be optimized. Metrics can be defined either on image level or at CV algorithm level. Tuning of ISP for human viewing is traditionally done subjectively based on aggregation of preferential scores of various test subjects. There are objective image quality metrics as well namely Mean Squared Error (MSE), Structural Similarity (SSIM), etc., but they may not correspond to perceptual similarity in general. For tuning CV algorithms, it is better to tune the KPIs of the algorithms directly. Typically, there will be multiple applications like Deep Learning, Structure from Motion, Visual SLAM, etc., The various accuracies of the individual algorithms have to be scaled via a weighted sum. The main challenge in tuning multiple algorithm KPIs is the time complexity of each iteration which may become impractical for large parameter search spaces. Thus some efficient strategies have to be employed. Image quality metrics can be tuned first to provide good initialization for the CV algorithm tuning method. It is worth nothing that in the case of deep learning algorithms, the ISP component may not be needed at all as the deep learning network can implicitly learn the necessary transformation. However, it may be better to explicitly model it within a deep learning network to exploit prior knowledge of imaging. Diamond et al. [94] use this idea to model a differentiable ISP module which can be trained end to end along with a recognition network.

Once a tuning metric and strategy are fixed, an optimization algorithm will find the best parameter set. The naive method is manual trial and error where an expert who developed the algorithm tunes the parameter set based on experience. Typically, a standard parameter set used in the CV community is used as a starting point which is refined further. In general, this is a tedious manual process and does not systematically yield optimal parameter set. Manual tuning is especially difficult for large search spaces of ISP with hundreds of parameters. The simplest optimization algorithms are grid search and random search. In grid search, parameter ranges are defined on a grid based on all possible combinations which are evaluated using brute force. For large parameter combinations, this is not practical. An improved approach is random search in which parameters are randomly chosen with a smart sampling strategy. It was demonstrated successfully in [95] for a large search space. However, convergence to an optimal parameter set is not guaranteed.

Recently, there are many smarter optimization approaches which are suitable for large search space problems. Bayesian Optimization [96] is a commonly used formal method for optimizing large-scale problems especially with computationally expensive evaluation functions. In principle, it can potentially absorb other search heuristic methods like genetic algorithms or swarm optimization techniques into a prior model. Thus it is a more generic and powerful approach. Formally, the problem can be defined as follows. An algorithm has a parameter space $P=\{p_{1},p_{2},\ldots ,p_{n}\}$ which is a set comprising of all possible parameter configurations. The parameters are typically numerical for e.g., [0.1,0.2,…,1.0] or can be categorical like enabling/disabling a module. The resolution and range of each parameter will be a key design choice. For ISP configuration with hundreds of parameters, the cardinality of P can exceed $10^{20}$ and a brute-force search is unfeasible.

The algorithm tuning problem can be defined as follows: $p_{opt}={\mathrm{argmax}}_{\forall p_{i}\in P}\left(F_{accuracy}\left(p\right)\right)$ where $p_{opt}$ is the optimal parameter configuration which maximizes the accuracy cost function $F_{accuracy}\left(p\right)$. Typically, there will be multiple algorithms whose accuracy’s have to be jointly optimized. The standard approach is to scalarize the different accuracies via a weighted sum. There are multi-objective optimization methods which aim to optimize KPIs simultaneously in multi-dimensional space. Typically, this is relatively complex and needs a lot of manual intervention too. Sequential Model-based Algorithm Configuration (SMAC3) (https://github.com/automl/SMAC3) is a popular tool for optimizing configuration and uses a combination of Bayesian optimization, gradient descent and other heuristics.

#### One ISP vs. Dual ISP

**Figure 19:**
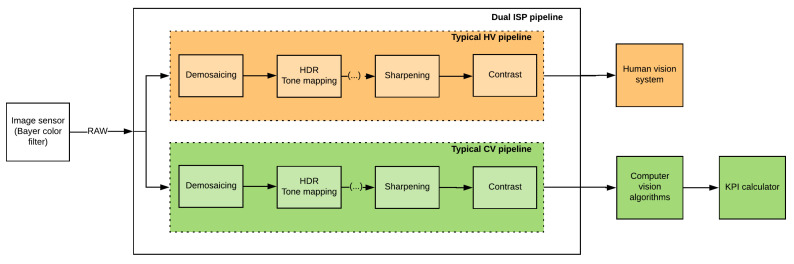
Illustration of Dual ISP.

The requirements can be different for human vision (HV) and computer vision (CV). Traditionally, due to cost, there was only one ISP either on board the processing SOC or a companion chip and this was typically tuned for human vision. Images from the same pipeline were used for machine vision applications. It has been demonstrated that ISPs optimized for HV and CV are different [94,97]. We also show additional empirical evidence of this in the next section. Splitting the ISP to have separate pipelines for both HV and CV would allow an independent tuning of each in order to independently maximize performance. This is particularly relevant for machine learning algorithms which are ideally trained on specific ISP settings. The dual ISP pipeline is illustrated in Figure 19. The importance of separate ISP has been understood better and most automotive SOCs now offer the compute power and memory bandwidth to enable dual ISP pipelines. Despite a multiple ISP implementation, there is a key restriction that dynamic feedback loop algorithms have to be controlled by one master. The low level behaviours of the corresponding image sensor, e.g., the exposure time at a pixel for a particular exposure, must be uniquely controlled.

## 5. Conclusions

A key trend in automotive systems is the move toward fully autonomous vehicles, and computer vision is no exception. All major vehicle manufacturers are investigating and promoting some form of vehicle autonomy, and all major manufacturers are investing in computer vision, with particular recent emphasis on neural networks. This has started with lower automation level applications already available on the market but will inevitably reach full fruition in future decades with fully autonomous vehicles. As such, cameras are, and will continue to be, a critical element in such systems.

The role of ISP in computer vision is critical, as it fundamentally controls the quality of the signal delivered to the computer vision algorithms. However, as we have discussed, “quality” for computer vision is not necessarily a well defined concept, particularly considering the vast range of algorithms and applications can be designed in computer vision. What is clear, as we have presented, is that maximising the performance of computer vision is critical in the autonomous vehicle context, and that adapting the ISP has a significant impact on the performance of computer vision algorithms. Given the importance and complexity of the topic, we have discussed here several possibilities around the automated tuning of ISP pipeline parameters using computer vision performance as a cost metric, such as Bayesian hyperparameter search, thus bypassing the need for a quality metric for computer vision on the image data that is delivered to the algorithm.

In this paper, we have presented what is predominantly an argument on the importance of automated tuning of image signal processing to maximise the performance of computer vision algorithms, presenting results to augment our argument but not presenting results on the automated tuning itself. This is clearly the next step in work in this direction. Related to the results presented in this study, the investigation will be widened to other ISP processes such as HDR, tone mapping, low light sensitivity, MTF and bit depth and to investigate the impact on KPIs of other CV, and more specifically DL, algorithms to optimally tune the ISP through automation as described.

## Figures and Tables

**Table 1:** KPI (%) given by PD algorithm after sharpening using Laplacian filters or unsharp masking (USM) or no filters (Original). TP = True Positive, FP = False Positive.

	Original (KPI)	Lap4 (KPI)	Lap8 (KPI)	USM3 (KPI)	USM9 (KPI)	USM19 (KPI)
Total results						
TP rate (%)	74.38	81.73	88.81	72.1	71.18	72.77
Total results						
FP per frame	0.1	0.109	0.118	0.091	0.090	0.095

**Table 2:** KPI (%) given by PD algorithm after contrast enhancement using CLAHE filters (CLAHE 2_8: CLAHE with clip limit = 2 & tile size = 8 × 8). TP = True Positive, FP = False Positive.

	Original (KPI)	CLAHE 2_8 (KPI)	CLAHE 2_16 (KPI)	CLAHE 10_8 (KPI)	CLAHE 10_16 (KPI)	CLAHE 40_8 (KPI)	CLAHE 40_16 (KPI)
Total results							
TP rate (%)	74.38	81.01	84.39	74.08	76.17	68.95	64.48
Total results							
FP per frame	0.1	0.11	0.098	0.062	0.043	0.042	0.022

**Table 3:** TP rate, FP rate and compromise values for original catalog, best configuration by TP rate and best configuration by compromise value.

	Original	Best Config (by $r_{\mathrm{TP}}$)	Best Config (by Γ)
Γ	0.7451	0.7589	0.7595
$r_{TP}$	0.7869	0.83	0.81
$r_{FP}$	0.055	0.095	0.069

**Table 4:** Parameters of the best configurations.

	Best Config (by $r_{\mathrm{TP}}$)	Best Config (by Γ)
Lap	Lap4	Lap8
clipLimit	2	2
tileSize	8 × 8	8 × 8

## Abbreviations

The following abbreviations are used in this manuscript:

AD	Autonomous Driving
ADAS	Advanced Driving Assistance System
ADC	Analog-to-Digital Conversion
AHE	Adaptive Histogram Equalization
AKAZE	Accelerated-KAZE
AWB	Auto White Balance
BRIEF	Binary Robust Independent Elementary Features
CLAHE	Contrast Limited Adaptive Histogram Equalization
CNN	Convolutional Neural Networks
CV	Computer Vision
DL	Deep Learning
DoG	Difference-of-Gaussian
FAST	Features from Accelerated Segment Test
FOV	Field Of View
FUN	Fidelity, Utility and Naturalness
HDR	High Dynamic Range
HE	Histogram Equalization
HOG	Histogram of Oriented Gradients
HV	Human Vision
ISP	Image Signal Processing/Processor/Processed, depending on context
KPI	Key Performance Indicators
LoG	Laplacian-of-Gaussian
LBP	Local Binary Patterns
MSE	Mean Square Error
MTF	Modulation Transfer Function
OEM	Original Equipment Manufacturer (in our context, the car manufacturers)
ORB	Oriented FAST and Rotated BRIEF
PD	Pedestrian Detection
RANSAC	RANdom SAmple Consensus
R-CNN	Regional-CNN
RGB	Additive color model in which the primary additive colours are red, green and blue
SfM	Structure of Motion
SIFT	Scale-Invariant Feature Transform
SLAM	Simultaneous Localization And Mapping
SMAC	Sequential Model-based Algorithm Configuration
SNR	Signal-to-Noise Ratio
SoC	System on Chip
SSIM	Structural SIMilarity
SURF	Speeded Up Robust Features
SVM	Support Vector Machine
TOF	Time Of Flight
TOPS	Tera Operations Per Second
USB	Universal Serial Bus
USM	Unsharp Masking
YUV	Colour space in terms of one luma (Y) and two chrominance (UV) components

## References

[1] Bovik A.C. (2013). Automatic prediction of perceptual image and video quality. Proc. IEEE.

[2] ITU-R Study Group (2012). BT.500: Methodology for the Subjective Assessment of the Quality of Television Pictures.

[3] ITU-T Study Group (2008). P.910: Subjective Video Quality Assessment Methods for Multimedia Applications.

[4] Hertel D.W., Chang E. Image quality standards in automotive vision applications. Proceedings of the 2007 IEEE Intelligent Vehicles Symposium.

[5] Winterlich A., Zlokolica V., Denny P., Kilmartin L., Glavin M., Jones E. A saliency weighted no-reference perceptual blur metric for the automotive environment. Proceedings of the 2013 Fifth International Workshop on Quality of Multimedia Experience (QoMEX).

[6] Heimberger M., Horgan J., Hughes C., McDonald J., Yogamani S. (2017). Computer vision in automated parking systems: Design, implementation and challenges. Image Vis. Comput..

[7] Johnson J. (1958). Analysis of image forming systems. Image Intensifier Symposium, AD220160.

[8] Velichko S. Intelligent Sensors For Autonomous Driving. Proceedings of the Automotive Forum of International Solid-State Circuits Conference (ISSCC).

[9] Gomez A. ISP Optimization for ML/CV Automotive Applications. Proceedings of the AutoSens Conference.

[10] Somayaji M. Tuning Image Processing Pipelines for Automotive Use. Proceedings of the AutoSens Conference.

[11] Yahiaoui L., Hughes C., Horgan J., Deegan B., Denny P., Yogamani S. Optimization of ISP parameters for object detection algorithms. Proceedings of the Electronic Imaging, Autonomous Vehicles and Machines Conference.

[12] Denny P., Jones E., Glavin M., Hughes C., Deegan B., Chen J., Cranton W., Fihn M. (2016). Imaging for the Automotive Environment. Handbook of Visual Display Technology.

[13] Girod B., Watson A.B. (1993). What’s wrong with mean-squared error? Digital Images and Human Vision.

[14] Eskicioglu A., Fisher P. (1995). Image quality meaures and their performance. IEEE Trans. Commun..

[15] Mannos J., Sakrison D. (1974). The effects of a visual fidelity criterion on the encoding of images. IEEE Trans. Inf. Theory.

[16] Eckert M., Bradley A. (1998). Perceptual quality metrics applied to still image compression. Signal Process..

[17] Wang Z., Bovick A. (2002). A universal image quality index. IEEE Signal Process. Lett..

[18] Wang Z., Bovik A., Sheikh H., Simoncelli E. (2004). Image Quality Assessment: From Error Visibility to Structural Similarity. IEEE Trans. Image Process..

[19] Wang Z., Simoncelli E., Bovik A. Multiscale structural similarity for image quality assessment. Proceedings of the Conference on Signals, Systems and Computers.

[20] Sheikh H., Bovik A., DeVeciana G. (2005). An information fidelity criterion for image quality assessment using natural scene statistics. IEEE Trans. Image Process..

[21] Sheikh H., Bovik A. (2006). Image information and visual quality. IEEE Trans. Image Process..

[22] Chandler D., Hemami S. (2007). A wavelet-based visual signal-to-noise ratio for natural images. IEEE Trans. Image Process..

[23] Zhang R., Isola P., Efros A. Colorful image colorization. Proceedings of the European Conference on Computer Vision (ECCV).

[24] Moorthy A., Bovik A. (2011). Blind image quality assessment: From natural scene statistics to perceptual quality. IEEE Trans. Image Process..

[25] Mittal A., Moorthy A., Bovik A. (2012). No-reference image quality assessment in the spatial domain. IEEE Trans. Image Process..

[26] Kingdom F., Prins N. (2016). Psychophysics: A Practical Introduction.

[27] Lu Z.L., Dosher B. (2013). Visual Psychophysics: From Laboratory to Theory.

[28] RichardWebster B., Anthony S.E., Scheirer W.J. (2019). PsyPhy: A Psychophysics Driven Evaluation Framework for Visual Recognition. IEEE Trans. Pat. Anal. Mach. Intel..

[29] Winterlich A., Denny P., Kilmartin L., Glavin M., Jones E. (2014). Performance optimization for pedestrian detection on degraded video using natural scene statistics. J. Electron. Imaging.

[30] Pezzementi Z., Tabor T., Yim S., Chang J., Drozd B., Guttendorf D., Wagner M., Koopman P. Putting image manipulations in context: Robustness testing for safe perception. Proceedings of the IEEE International Symposium on Safety, Security, and Rescue Robotics (SSRR).

[31] Su J., Vargas D.V., Sakurai K. (2019). One pixel attack for fooling deep neural networks. IEEE Trans. Evolut. Comput..

[32] Szegedy C., Zaremba W., Sutskever I., Bruna J., Erhan D., Goodfellow I.J., Fergus R. (2014). Intriguing properties of neural networks. arXiv.

[33] Buckler M., Jayasuriya S., Sampson A. Reconfiguring the imaging pipeline for computer vision. Proceedings of the IEEE International Conference on Computer Vision (ICCV).

[34] Lowe D.G. (2004). Distinctive Image Features from Scale-Invariant Keypoints. Int. J. Comput. Vision.

[35] Blasinski H., Farrell J., Lian T., Liu Z., Wandell B. Optimizing Image Acquisition Systems for Autonomous Driving. Proceedings of the Electronic Imaging, Photography, Mobile, and Immersive Imaging Conference.

[36] Young I.T. (2001). Shading correction: compensation for illumination and sensor inhomogeneities. Curr. Protoc. Cytom..

[37] Yung-Cheng Liu W.H.C., Chen Y.Q. (1995). Automatic white balance for digital still camera. IEEE Trans. Consum. Electron..

[38] SangHyun Park G.K., Jeon J. The method of auto exposure control for low-end digital camera. Proceedings of the 11th International Conference on Advanced Communication Technology.

[39] Fowler K.R. (2004). Automatic gain control for image-intensified camera. IEEE Trans. Instrum. Meas..

[40] Yuan X., He P., Zhu Q., Bhat R.R., Li X. (2019). Adversarial Examples: Attacks and Defenses for Deep Learning. IEEE Trans. Neur. Net. Lear..

[41] Yoo Y., Lee S.D., Choe W., Kim C.Y. CMOS image sensor noise reduction method for image signal processor in digital cameras and camera phones. Proceedings of the Digital Photography.

[42] Losson O., Macaire L., Yang Y. (2010). Comparison of Color Demosaicing Methods. Adv. Imag. Electron. Phys..

[43] Takahashi K., Monno Y., Tanaka M., Okutomi M. Effective color correction pipeline for a noisy image. Proceedings of the 2016 IEEE International Conference on Image Processing (ICIP).

[44] Morris T. (2004). Computer Vision and Image Processing.

[45] Horgan J., Hughes C., McDonald J., Yogamani S. Vision-based driver assistance systems: Survey, taxonomy and advances. Proceedings of the 2015 IEEE 18th International Conference on Intelligent Transportation Systems.

[46] Hartley R., Zisserman A. (2003). Multiple View Geometry in Computer Vision.

[47] Tareen S.A.K., Saleem Z. A comparative analysis of SIFT, SURF, KAZE, AKAZE, ORB, and BRISK. Proceedings of the 2018 International Conference on Computing, Mathematics and Engineering Technologies (iCoMET).

[48] Tang Z., Boukerche A. An Improved Algorithm for Road Markings Detection with SVM and ROI Restriction: Comparison with a Rule-Based Model. Proceedings of the International Conference on Communications (ICC).

[49] Li L., Luo W., Wang K. (2018). Lane Marking Detection and Reconstruction with Line-Scan Imaging Data. Sensors.

[50] Jung H.G., Kim D.S., Yoon P.J., Kim J. Parking slot markings recognition for automatic parking assist system. Proceedings of the 2006 IEEE Intelligent Vehicles Symposium.

[51] Liu Y.C., Lin K.Y., Chen Y.S. Bird’s-eye view vision system for vehicle surrounding monitoring. Proceedings of the International Conference on Robot Vision (RobVis).

[52] Jung H.G., Kim D.S., Yoon P.J., Kim J. Structure Analysis Based Parking Slot Marking Recognition For Semi-Automatic Parking System. Proceedings of the Joint IAPR International Workshops on Statistical Techniques in Pattern Recognition (SPR) and Structural and Syntactic Pattern Recognition.

[53] Su B., Lu S. A System for Parking Lot Marking Detection. Proceedings of the Pacific-Rim Conference on Multimedia (PCM).

[54] Geiger A., Lenz P., Stiller C., Urtasun R. (2013). Vision meets robotics: The KITTI dataset. Int. J. Robot. Res..

[55] Chavan A., Yogamani S.K. Real-time DSP implementation of pedestrian detection algorithm using HOG features. Proceedings of the 12th International Conference on ITS Telecommunications.

[56] Siam M., Gamal M., Abdel-Razek M., Yogamani S., Jagersand M. RTSeg: Real-time semantic segmentation comparative study. Proceedings of the 25th IEEE International Conference on Image Processing (ICIP).

[57] Ilg E., Mayer N., Saikia T., Keuper M., Dosovitskiy A., Brox T. (2016). Flownet 2.0: Evolution of optical flow estimation with deep networks. arXiv.

[58] Siam M., Mahgoub H., Zahran M., Yogamani S., Jagersand M., El-Sallab A. Modnet: Motion and appearance based moving object detection network for autonomous driving. Proceedings of the 2018 21st International Conference on Intelligent Transportation Systems (ITSC).

[59] Kumar V.R., Milz S., Witt C., Simon M., Amende K., Petzold J., Yogamani S., Pech T. Monocular fisheye camera depth estimation using sparse lidar supervision. Proceedings of the 2018 21st International Conference on Intelligent Transportation Systems (ITSC).

[60] Milz S., Arbeiter G., Witt C., Abdallah B., Yogamani S. Visual SLAM for Automated Driving: Exploring the Applications of Deep Learning. Proceedings of the IEEE Conference on Computer Vision and Pattern Recognition Workshops.

[61] Uricar M., Krizek P., Sistu G., Yogamani S. SoilingNet: Soiling Detection on Automotive Surround-View Cameras. Proceedings of the IEEE Intelligent Transportation Systems Conference.

[62] Sistu G., Leang I., Chennupati S., Milz S., Yogamani S., Rawashdeh S. NeurAll: Towards a Unified Model for Visual Perception in Automated Driving. Proceedings of the IEEE Intelligent Transportation Systems Conference.

[63] Valada A., Oliveira G.L., Brox T., Burgard W. Deep Multispectral Semantic Scene Understanding of Forested Environments using Multimodal Fusion. Proceedings of the International Symposium on Experimental Robotics (ISER).

[64] Bonanni T.M., Pennisi A., Bloisi D., Iocchi L., Nardi D. Human-robot collaboration for semantic labeling of the environment. Proceedings of the 3rd Workshop on Semantic Perception, Mapping and Exploration.

[65] Vineet V., Miksik O., Lidegaard M., Nießner M., Golodetz S., Prisacariu V.A., Kähler O., Murray D.W., Izadi S., Perez P. Incremental Dense Semantic Stereo Fusion for Large-Scale Semantic Scene Reconstruction. Proceedings of the IEEE International Conference on Robotics and Automation (ICRA).

[66] Kundu A., Li Y., Dellaert F., Li F., Rehg J.M. Joint semantic segmentation and 3d reconstruction from monocular video. Proceedings of the European Conference on Computer Vision.

[67] Çiçek Ö., Abdulkadir A., Lienkamp S.S., Brox T., Ronneberger O. 3D U-Net: Learning dense volumetric segmentation from sparse annotation. Proceedings of the International Conference on Medical Image Computing and Computer-Assisted Intervention.

[68] Zhu W., Xie X. (2016). Adversarial deep structural networks for mammographic mass segmentation. arXiv.

[69] Miksik O., Vineet V., Lidegaard M., Prasaath R., Nießner M., Golodetz S., Hicks S.L., Pérez P., Izadi S., Torr P.H. The semantic paintbrush: Interactive 3d mapping and recognition in large outdoor spaces. Proceedings of the 33rd Annual ACM Conference on Human Factors in Computing Systems.

[70] Siam M., Elkerdawy S., Jagersand M., Yogamani S. Deep semantic segmentation for automated driving: Taxonomy, roadmap and challenges. Proceedings of the IEEE 20th International Conference on Intelligent Transportation Systems (ITSC).

[71] Farabet C., Couprie C., Najman L., LeCun Y. (2013). Learning hierarchical features for scene labeling. IEEE Trans. Pattern Anal. Mach. Intell..

[72] Farabet C., Couprie C., Najman L., Lecun Y. Scene Parsing with Multiscale Feature Learning, Purity Trees, and Optimal Covers. Proceedings of the 29th International Conference on Machine Learning.

[73] Grangier D., Bottou L., Collobert R. Deep convolutional networks for scene parsing. Proceedings of the ICML 2009 Deep Learning Workshop.

[74] Long J., Shelhamer E., Darrell T. Fully convolutional networks for semantic segmentation. Proceedings of the IEEE Conference on Computer Vision and Pattern Recognition (CVPR).

[75] Noh H., Hong S., Han B. Learning deconvolution network for semantic segmentation. Proceedings of the IEEE International Conference on Computer Vision.

[76] Badrinarayanan V., Kendall A., Cipolla R. (2017). SegNet: A Deep Convolutional Encoder-Decoder Architecture for Image Segmentation. IEEE Trans. Pattern Anal. Mach. Intell..

[77] Yu F., Koltun V. Multi-scale context aggregation by dilated convolutions. Proceedings of the International Conference on Learning Representations (ICLR).

[78] Chen L.C., Yang Y., Wang J., Xu W., Yuille A.L. Attention to scale: Scale-aware semantic image segmentation. Proceedings of the IEEE Conference on Computer Vision and Pattern Recognition (CVPR).

[79] Qi G.J. Hierarchically Gated Deep Networks for Semantic Segmentation. Proceedings of the IEEE Conference on Computer Vision and Pattern Recognition (CVPR).

[80] Ronneberger O., Fischer P., Brox T. U-net: Convolutional networks for biomedical image segmentation. Proceedings of the International Conference on Medical Image Computing and Computer-Assisted Intervention.

[81] Ilg E., Mayer N., Saikia T., Keuper M., Dosovitskiy A., Brox T. Flownet 2.0: Evolution of optical flow estimation with deep networks. Proceedings of the IEEE Conference on Computer Vision and Pattern Recognition (CVPR).

[82] Ummenhofer B., Zhou H., Uhrig J., Mayer N., Ilg E., Dosovitskiy A., Brox T. DeMoN: Depth and motion network for learning monocular stereo. Proceedings of the IEEE Conference on Computer Vision and Pattern Recognition (CVPR).

[83] Borkar T.S., Karam L.J. (2019). DeepCorrect: Correcting DNN models against image distortions. IEEE Trans. Image Process..

[84] Dodge S.F., Karam L.J. Understanding How Image Quality Affects Deep Neural Networks. Proceedings of the International Conference on Quality of Multimedia Experience (QoMEX).

[85] Winterlich A., Hughes C., Kilmartin L., Glavin M., Jones E. (2015). An oriented gradient based image quality metric for pedestrian detection performance evaluation. Signal Process. Image Commun..

[86] Yahiaoui L., Horgan J., Yogamani S., Hughes C., Deegan B. Impact Analysis and Tuning Strategies for Camera Image Signal Processing Parameters in Computer Vision. Proceedings of the Irish Machine Vision and Image Processing Conference (IMVIP).

[87] Shrivakshan G., Chandrasekar C. (2012). A Comparison of various Edge Detection Techniques used in Image Processing. Int. J. Comput. Sci. Issues.

[88] Haralick R.M., Sternberg S.R., Zhuang X. (1987). Image Analysis Using Mathematical Morphology. IEEE Trans. Pattern Anal. Mach. Intell..

[89] Bay H., Ess A., Tuytelaars T., Gool L.V. (2008). Speeded-Up Robust Features (SURF). Comput. Vis. Image Underst..

[90] Rublee E., Rabaud V., Konolige K., Bradski G. ORB: An efficient alternative to SIFT or SURF. Proceedings of the International Conference on Computer Vision (ICCV).

[91] Alcantarilla P.F., Bartoli A., Davison A.J. KAZE Features. Proceedings of the European Conference on Computer Vision (ECCV).

[92] Alcantarilla P.F., Nuevo J., Bartoli A. (2013). Fast Explicit Diffusion for Accelerated Features in Nonlinear Scale Spaces. IEEE Trans. Patt. Anal. Mach. Intell..

[93] Pizer S.M., Amburn E.P., Austin J.D., Cromartie R., Geselowitz A., Greer T., ter Haar Romeny B., Zimmerman J.B., Zuiderveld K. (1987). Adaptive histogram equalization and its variations. Comput. Vision Graph. Image Process..

[94] Diamond S., Sitzmann V., Boyd S., Wetzstein G., Heide F. (2017). Dirty pixels: Optimizing image classification architectures for raw sensor data. arXiv.

[95] Bergstra J., Bengio Y. (2012). Random search for hyper-parameter optimization. J. Mach. Learn. Res..

[96] Snoek J., Larochelle H., Adams R.P. Practical bayesian optimization of machine learning algorithms. Proceedings of the 25th International Conference on Neural Information Processing Systems (NIPS).

[97] Blau Y., Michaeli T. The Perception-Distortion Tradeoff. Proceedings of the Conference on Computer Vision and Pattern Recognition (CVPR).

